# Development and characterization of various osteoarthritis models for tissue engineering

**DOI:** 10.1371/journal.pone.0194288

**Published:** 2018-03-13

**Authors:** Ji Eun Kim, Da-hyun Song, Soo Hyun Kim, Youngmee Jung, Sang Jun Kim

**Affiliations:** 1 KU-KIST Graduate School of Converging Science and Technology, Korea University, Seoul, Korea; 2 Center for Biomaterials, Korea Institute of Science and Technology, Seoul, Republic of Korea; 3 Department of Physical and Rehabilitation Medicine, Samsung Medical Center, Seoul, Republic of Korea; 4 Department of Biomedical Engineering, Korea University of Science and Technology (UST), Daejeon, Republic of Korea; University of Umeå, SWEDEN

## Abstract

Osteoarthritis (OA) is characterized by a progressive loss of articular cartilage, subchondral bone sclerosis and synovial inflammation and is the most common chronic condition worldwide today. However, most treatments have focused on pain relief and OA symptoms. For these reasons, many ongoing studies are currently trying to develop efficient and successful therapies based on its pathology. Animal models that mimic the histopathology and symptoms of OA have a critical role in OA research and make it possible to investigate both secondary osteoarthritic changes due to a precedent event such as traumatic injury and naturally occurring changes for the development of therapeutics which can be tested in preclinical and clinical OA trials. We induced OA in various animal models including rats, rabbits and guinea pigs by chemical, surgical and naturally occurring methods. In particular, the Dunkin-Hartley guinea pig is very attractive as an OA animal model because OA slowly progresses which is similar to human primary OA. Thus, this animal model mimics the pathophysiological process and environment of human primary OA. Besides the spontaneous OA model, anterior cruciate ligament transection (ACLT) with medial meniscectomy and bilateral ovariectomy (OVX) as well as a chemical technique using sodium monoiodoacetate (MIA) were used to induce OA. We found that ACLT in the rat model induced OA changes in the histology and micro-CT image compared to OVX. The osteoarthritic change significantly increased following ACLT surgery in the rabbit model. Furthermore, we identified that OA pathogenic changes occurred in a time-dependent manner in spontaneous Dunkin-Hartley guinea pigs. The MIA injection model is a rapid and minimally invasive method for inducing OA in animal models, whereas the spontaneous OA model has a slow and gradual progression of OA similar to human primary OA. We observed that histological OA change was extraordinarily increased at 9 ½ months in the spontaneous OA model, and thus, the grade was similar with that of the MIA model. Therefore, this study reports on OA pathology using various animal models as well as the spontaneous results naturally occurring in an OA animal model which had developed cartilage lesions and progressive osteoarthritic changes.

## Introduction

Osteoarthritis (OA) is a degenerative arthritis disease caused by overweight, injury, genes and other factors [1–3]. Recently, aging has become the most common cause of OA which can induce chronic joint pain, excessive morbidity and a progressive loss of extracellular matrices (ECMs) in joint cartilage and bone [4–6]. Articular cartilage degradation results from biochemical alterations which are associated with not only structural and metabolic changes but also with imbalances between synthetic and degradative pathways [7]. Many preclinical and clinical trials have been done attempting to alleviate or delay OA progression; however, no suitable treatments have been found that halt the progression of the disease [8].

Generally, OA is classified into primary and secondary OA. Primary OA is a naturally occurring disease resulting from senile changes and is a generalized disorder while secondary OA is caused by localized lesions such as congenital, acute trauma and other disorders of the bone. Because primary OA affects all parts of an articular joint unlike secondary OA localized to an injured area, its clinical therapy is focused on the inhibition of degenerative progression including autologous grafts and chondrocyte implantation rather than surgical repair [9,10].

To find efficacious treatments, numerous OA animal models have been investigated with various methods. These models are also needed to investigate the pathogenesis of OA and the therapeutic efficacy of new treatment modalities [11]. Currently, OA models have only been induced in 10 different species of varying strain, age, and gender with a variety of induction methods [12]. Many animal species and many OA induction models have been used to simulate the human OA model. *Mouse*, rat, rabbit, guinea pig and large animals have been studied for the OA model. Induction of the OA model has been accomplished through genetic modification (STR/ort mice) [13,14], surgical method (anterior cruciate ligament transection, ACLT [15–17]/ medial meniscectomy; MM [18,19]/ bilateral ovariectomy; OVX [20]), intra-articular injection (monosodium iodoacetate; MIA/ collagenase, papain) [21–24] and spontaneous model without any manipulation. In addition, groove model has been studied for developed OA animal model. This model leads to mechanical damage of the articular cartilage followed by transient forced loading of the surgically defected joint surface. Rats and canines have been used to study the groove model to induce degenerative changes in their knee [25–27]. ACLT model is the most frequently used for surgical OA model, which occurs the permanent instability in the articular cartilage knee joint. Compared to ACLT, the local damage lesions in groove model develop according to temporary triggers. For these reasons, groove model has advantages which are possible to deduce the sensitive results and long-term follow up after clinical treatment of OA because there is no continuous trigger [27]. However, there has been no gold standard model that simulates human OA because each model and each species have their own merits.

To select an OA animal model for OA research, the following parameters must be considered: animal care, costs, ease of handling, disease progression, and response to specific treatments [2]. For example, the ACLT model is most commonly used for an OA induced animal model because the results are highly reproducible, and OA rapidly progress [28–30]. Furthermore, this method results in molecular changes in the cartilage, synovial inflammation and subchondral bone sclerosis like human OA [31]. For these reasons, this surgical OA model is suitable for short-term studies. The ovariectomy model mimics postmenopausal human OA well; however, it does not mimic traumatic changes in the cartilage and bone which occur in human OA [32]. In addition to the surgically induced OA model, chemically induced models investigate a compound by direct injection into the knee joint. For these reasons, chemical injection models are easy to induce and have the least costs; however, they do not accurately mimic the chronic progression of OA. MIA is the typical compound used for the OA chemical induction animal model which is known to inhibit the glyceraldehyde-3-phosphate dehydrogenase activity. The inhibition of glycolysis by MIA leads to not only chondrocyte death but also articular cartilage degradation [23,33]. Furthermore, some chemical reagents such as papain, quinolone and collagenase are also known to induce OA in animal models [30]. However, decreases in the proteoglycan matrix of the cartilage as well as functional joint impairments can be induced by MIA administration into rodent articular cartilage which have similar characteristics with human OA [34].

Compared to the above-mentioned induced models, spontaneous models have the different stages of primary OA which not only naturally occur but also progress slowly simulating OA. These models have advantages that rely on pathological changes rather than post-traumatic alterations; however, they require much time and high cost to make. Dunkin-Hartley guinea pigs usually develop spontaneous OA because the pathophysiological processes including slow progressive changes in them closely mimic human OA [28,35,36].

Many researchers have investigated the process of spontaneous OA based on age-related changes using various analytical tools including pathohistological, immunohistological, histomorphometric and radiologic techniques in Dunkin-Hartley guinea pigs. Jimenez et al. reported that the mild OA in guinea pigs was detected at 3 months of age by the presence of minimal histologic changes which is the mild stage of OA [37]. Similarly, Tokuda observed by histological changes that a decrease in chondrocytes in the knee joints began to appear at 3 months of age. Furthermore, at 5 months of age, fibrillation gradually occurred, and at 8 months of age, the incidence of fibrillation reached 100%. They suggested that OA severity significantly increases with age, and the cartilage thickness was significantly reduced at 18 months of age [38]. Therefore, this study induced numerous OA animal models using middle-sized animals that develop lesions resembling those of human OA for the development of therapeutics which can be tested in preclinical and clinical OA trials.

To determine the pathogenesis of OA, to investigate the therapeutic efficacy of new treatments, and to extrapolate preclinical results to clinical applications, selecting the optimal animal species and OA induction model is mandatory. In this study, we describe the characteristics of OA and the progressive changes within the articular cartilage of a knee joint with various evaluation processes to observe the pathogenesis of OA in diverse animal species using diverse OA induction methods and to extrapolate the preclinical results to clinical applications.

## Materials and methods

For the OA model, animals were anesthetized with an intraperitoneal injection of a 1:1 mixture of tiletamine and zolazepam (Zoletil 50; Virbac, Carros, France) with xylazine (Rompun; Bayer, Leverkusen, Germany) at a dose of 30 mg Zoletil and 10 mg Rompun per kilogram of body weight. In this study, all animal experiments were approved by the Institutional Animal Care and Use Committee at the Korea Institute of Science and Technology and Samsung medical center in accordance with the recommendations for handling laboratory animals for biomedical research.

### Rat ACLT surgery

**Fig 1:**
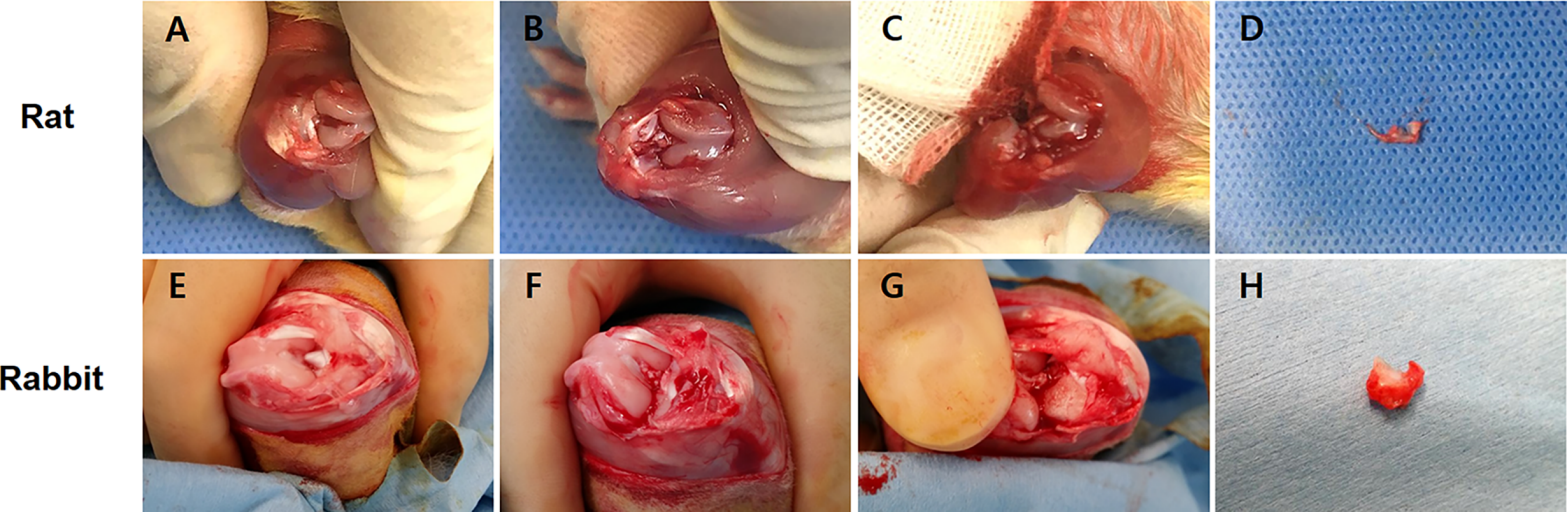
OA induction procedures for anterior cruciate ligament transection (ACLT) in the animal models. (A-D) The ACLT images for the rat animal model. (A) A vertical midline incision was made in the skin, followed by the elimination of the fat pad, and then, the anterior cruciate ligament (ACL) was exposed in the knee joint. (B) The ACL was dissected with a surgical scissor. (C) The medial meniscus was completely removed, and the medial collateral ligament (MCL) was transected too. (D) The image shows the extracted medial meniscus from the knee joint of rats. (E-H) The ACLT images for the rabbit animal model. (E) After a vertical midline incision, the patella was pushed laterally to expose the ACL. (F) The exposed ACL was cut in the rabbit model. (G) The medial meniscus was removed, and the MCL was transected without injury to the cartilage. (H) The image shows the completely extracted medial meniscus from the rabbit knee joint.

Nine Sprague Dawley (SD) female rats 15 weeks old underwent ACLT surgery to induce the OA model. The left hind limb was shaved and cleansed with povidone iodine. Fig 1. shows the OA induction procedures for ACLT in the rat animal models. The skin at the medial side of the knee cap was incised about 2 cm to expose the patella and patellar tendon. After displacing the patellar laterally with a curved forcep with the knee flexed, the joint capsule was exposed. The joint capsule was removed, and the ACL was cut with a sharp scissor. Complete resection of the ACL was confirmed by displacing the tibia from the femur anteriorly. Medial collateral ligament and medial meniscus were also completely cut to make the knee joint unstable. The patellar was moved back to the midline, and the fascia and skin were sutured sequentially by 3–0 polydioxanone and 3–0 nylon threads. The control group comprised rats that did not undergo OA by ACLT surgery.

### Rat ovariectomy surgery

**Fig 2:**
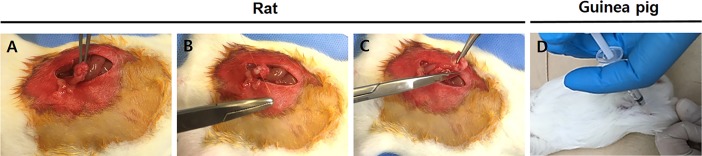
Images for the surgical and chemical induction procedures for the OA animal models. (A-C) Images of the bilateral ovariectomy (OVX) procedure in the rat animal model. (A) The ovary was exposed which is located in the tip of the uterine horn. (B) The ovarian duct was ligated at 1 cm distal to the ovary with a suture. (C) The ligated ovarian duct was cut and extracted by a surgical scissor. (D) To induce OA, sodium monoiodoacetate (MIA) was injected into the knee joint of a Dunkin-Hartley guinea pig.

Five female SD rats 15 weeks old underwent bilateral ovariectomy to induce the OA model. Fig 2. shows the OA induction procedures for OVX in the rat animal models. After the anesthetization, the rats were placed in the supine position, and about a 2 cm paramedian incision was done at the level of the lower abdomen. The abdominal muscle was put aside, and an ovary was picked up with a straight forcep. After ligation of the ovarian duct 1 cm proximal to the ovary, the ovary was cut and removed by a surgical scissor. Likewise, another ovariectomy was done for the other ovary. The control group comprised rats that did not undergo OA by bilateral ovariectomy surgery.

### Rabbit ACLT surgery

In this study, five New Zealand white female rabbits 16 weeks old were used for the ACLT surgery. The ACLT procedure for the rabbit OA model was the same as that for the rat OA model (Fig 1). Complete dissection of the ACL and MCL and complete resection of the medial meniscus were done after displacing the patella laterally. The fascia was sutured with polydioxane to prevent patellar subluxation. The control group comprised rabbits that did not undergo OA by ACLT surgery.

### Guinea pig MIA model injection

For the chemically induced OA model, six Dunkin-Hartley female guinea pigs 15 weeks old were used in this study. The guinea pigs were allowed to acclimate to the facility for 7 days. MIA (Bioworld) was dissolved in physiologic saline (100 μg/μL) and filtered with a syringe filer, and then, 100 μL were administered with a 26-gauge needle into the joint cavity of the right knee (Fig 2). Four weeks after the injection, the guinea pigs were sacrificed and examined to determine whether OA was induced by the administration of MIA. The control group is non-treated group as native model at 5 months.

### Guinea pig spontaneous model

Twelve Dunkin-Hartley female guinea pigs 15 weeks old were obtained for the naturally occurring OA model. They were housed two per cage. To observe the degenerative change of the knee joint according to age, the Dunkin-Hartley guinea pigs were sacrificed at 6^1/2^ 8, 9^1/2^ and 11 months.

### Micro-CT imaging study

The distal part of the femur and the proximal part of the tibia were cut with a blunt scissor to acquire knee joint tissues. Muscles and ligaments were completely dissected, and bone and cartilage were scanned with a micro-CT. The knee joints of the rats were imaged with a micro-CT scanner after a month following the OA surgical induction by ACLT and OVX. At 36 weeks post-surgical induction, the knee joints of the rabbits were extracted for the micro-CT imaging study. To observe the spontaneous osteoarthritic changes, the knee joints of the Dunkin Hartley guinea pigs were imaged at 6^1/2^ 8, 9^1/2^ and 11 months. Furthermore, guinea pigs were also sacrificed at 30 days post-MIA injection for the chemically induced OA model. The scanning time for the micro-CT scanner was adjusted to 0.21 seconds with a setting of 80 kVp, 500 μA, and 30 calibrations. A 30.74-mm axial and trans-axial fields of view were acquired. Micro-CT images were also reconstructed into a three-dimensional image to show the OA changes.

### Histology

The harvested knee joints of the rats, rabbits and guinea pigs were stained as described by Appleton et al. for histological analysis [39]. The collected samples were fixed in 10% (v/v) buffered formalin, decalcified by hydrogen chloride ethylenediaminetetraacetic acid solution (Sigma, Merck, Darmstadt, Germany), dehydrated in a graded ethanol series, and embedded in paraffin. Then, the specimens were sectioned in the sagittal plane under the midline at 6 μm thicknesses. To observe the nucleus and cytoplasm, we conducted hematoxylin and eosin (H&E) staining. Furthermore, the sulfated GAG in the retrieved constructs was stained with alcian blue and safranin O. Light microscope photographs of the stained slides were taken.

### Histological scoring system

A modified Mankin scoring system was used to evaluate the development of OA. Four items were checked: 1) cartilage structure (0–6); 2) cartilage cells (0–3); 3) Safranin O or Alcian blue staining (0–4), and 4) tidemark integrity (0–1). The total global score was the sum of these four scores.

### Statistical analysis

Quantitative results are expressed as the mean ± standard deviation with the Graphpad Prism software (Graphpad Prism, version 7; GraphPad Software Inc, CA, USA) to evaluate the correlation between the experimental groups. Statistical analysis was performed either with one-way analysis of variance (ANOVA) with Tukey’s post-tests or two-way ANOVA with Bonferroni post-tests. Significant difference was determined with the post-tests between the experimental groups. The P value was set at *P* < 0.05.

## Results

### Micro-CT imaging study

**Fig 3:**
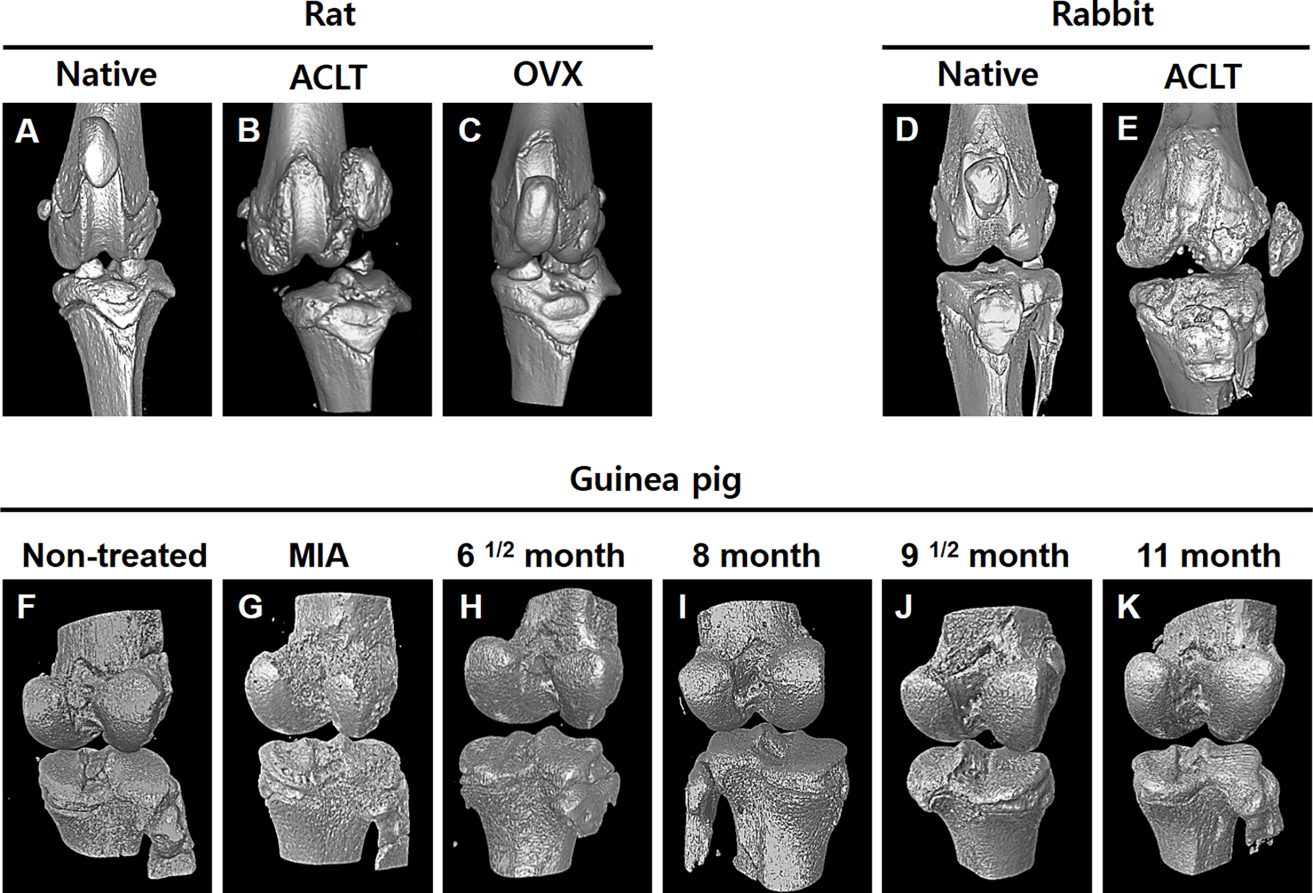
Micro-computed tomography (micro-CT) images of the knee joints from the OA animal models. **The micro-CT images were reconstructed to three-dimensional images.** (A-C) Images of the knee joints from the OA rat model induced by anterior cruciate ligament transection and bilateral ovariectomy. (D-E) Images of the knee joints from the OA rabbit model induced by anterior cruciate ligament transection. (F-K) Micro-CT images of the Dunkin-Hartley guinea pig OA knee joints. (G) Image of OA knee joints induced by sodium monoiodoacetate (MIA) chemical injection. (H-K) Micro-CT images of Dunkin-Hartley guinea pig knee joints according to age in the spontaneous OA model.

Micro-CT images of the rat, rabbit and guinea pig knees were obtained to characterize OA progression. Representative micro-CT 3D images of the OA rat model are shown in Fig 3A–3C. The knee joints of the ACLT group have a rough and irregular surface at the medial and lateral femur areas at 4 weeks post-surgery. The medial meniscus was completely eliminated for OA induced by ACLT surgery. In contrast, there were no obvious macroscopic changes in the native and OVX group. The OVX group had slight damage to the cartilage surface at the lateral femur region compared to that of the native group. To observe cartilage degeneration in the Dunkin-Hartley guinea pigs, the knee joints were examined with a micro-CT scanner one month after the MIA injection into the knee joint cavity. Compared with the non-treated knee joint specimens, the cartilage surface of the treated knee specimens shows severe loss and erosion of the cartilage in the medial tibial plateau and femur areas (Fig 3G). However, the macroscopic observation of the spontaneous model showed no changes at 8 months. Continuous changes by naturally occurring OA gradually appeared by 9^1/2^ months. As time progressed, the damaged regions of cartilage gradually expanded; however, the tendency for change was weak compared to the MIA treated group.

### Histology study

**Fig 4:**
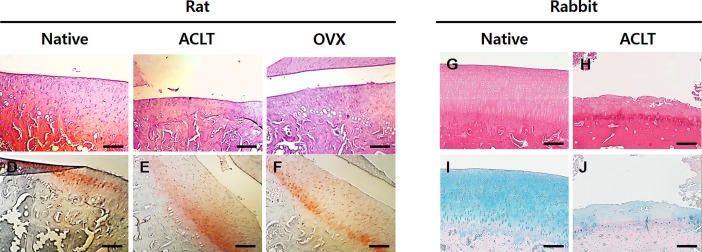
Histological studies of OA knee joints induced by surgical induction techniques. (A-C) Macroscopic images of hematoxylin and eosin staining of the rat animal model. (D-F) Macroscopic images of safranin O staining of the rat animal model. (G, H) Macroscopic images of hematoxylin and eosin staining of the rabbit animal model. (I, J) Macroscopic images of alcian blue staining of the rabbit animal model. (Scale bars: 200 μm).

Fig 4 shows the histological changes in OA severity in the knee joints of the rat and rabbit models. Well-formed cartilaginous tissues containing cytoplasm and nuclei were revealed in the rat native model. Furthermore, the native group had a smooth and regular cartilage surface. However, the H&E staining images of the ACLT group showed a thin layer of superficial cartilage with synovial tissues and the partial disappearance of cartilage cells. Moreover, the OVX group had some erosion at the cartilage surface; however, the severity was less than that of the ACLT group. Safranin O staining was done to examine the sulfated GAGs. There was a sufficient amount of GAGs in the rat knee joints of the native group. However, the ACLT and OVX group had incomplete cartilage which was weakly stained for GAGs compared to that of the native group.

Likewise, histological sections from the rabbit OA models were used to identify the cartilaginous structure and morphology of the cartilage cells containing cytoplasm and nuclei. Native tissues showed well-formed cartilaginous tissues and chondrocytes with clear round shapes. Furthermore, the specimens did not exhibit the typical OA characteristics such as the degeneration and depletion of chondrocytes and an irregular cartilage surface. In contrast, the specimens of the ACLT group had obvious OA characteristics such as the loss of chondrocytes, an extremely thin cartilage thickness and a damaged cartilage surface when compared with the native tissues. Alcian blue staining showed an abundant accumulation of GAGs in the rabbit native cartilage tissues. However, the rabbit specimens of the ACLT group were weakly stained for GAGs, and chondrocytes were almost lost in three zones including the superficial, middle and deep zone.

**Fig 5:**
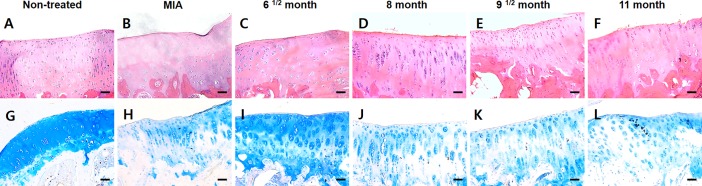
Histological studies of OA knee joints in Dunkin-Hartley guinea pigs. Hematoxylin and eosin staining (upper line) and alcian blue staining (lower line) of the retrieved knee joints from guinea pigs. (B, H) The stained image of OA knee joints induced by sodium monoiodoacetate (MIA) chemical injection. (C-F, I-L) The stained images of Dunkin-Hartley guinea pig knee joints according to age in the spontaneous OA model. (Scale bars: 100 μm).

Furthermore, the naturally and chemically induced guinea pig OA model exhibited histological changes in the cartilaginous tissues (Fig 5). The articular cartilage had a smooth surface and well-formed cartilaginous tissues in the non-treated group at 5 months. Although the cartilage was observed at the same time point, the MIA-treated knee joint had a partially caved in cartilage surface and loosely distributed chondrocytes. Histopathological changes in the naturally occurring models appear to occur sequentially and in a time-dependent manner. At 6^1/2^ months, there was a decreased distribution of chondrocytes in part of the superficial zone; however, the cartilage surface was comparatively smooth which was similar to the non-treated group. At 8 months, a rough and irregular surface was observed compared to that of the non-treated group. At 9^1/2^ months, the chondrocytes disappeared in articular cartilage with much less distribution of chondrocytes than that of the specimen at 8 months. Furthermore, it was observed that the region of chondrocyte loss was expanded from the transitional to the radial zone in the knee joint at 11 months; additionally, the depletion of cartilaginous tissues occurred in the adjacent region with the subchondral bone. In the non-treated group at 6 months, a sufficient amount of GAGs was well-distributed in the cartilage tissues. The specimens from the MIA treated knee joints had a slightly reduced stainability with alcian blue, and an irregular cartilage surface was observed. In addition, the spontaneous OA models in the guinea pigs were examined not only for severe OA in the knee but also for incomplete cartilage tissues based on alcian blue staining. The spontaneous OA model was weakly stained for GAGs, and surface-layer cells and chondrocytes disappeared in the transitional and radial zone as time progressed. Furthermore, at later time points, a few stained regions gradually expanded from the outside to the inside. This result means that severe OA developed and progressively increased destroying the cartilaginous tissues.

**Fig 6:**
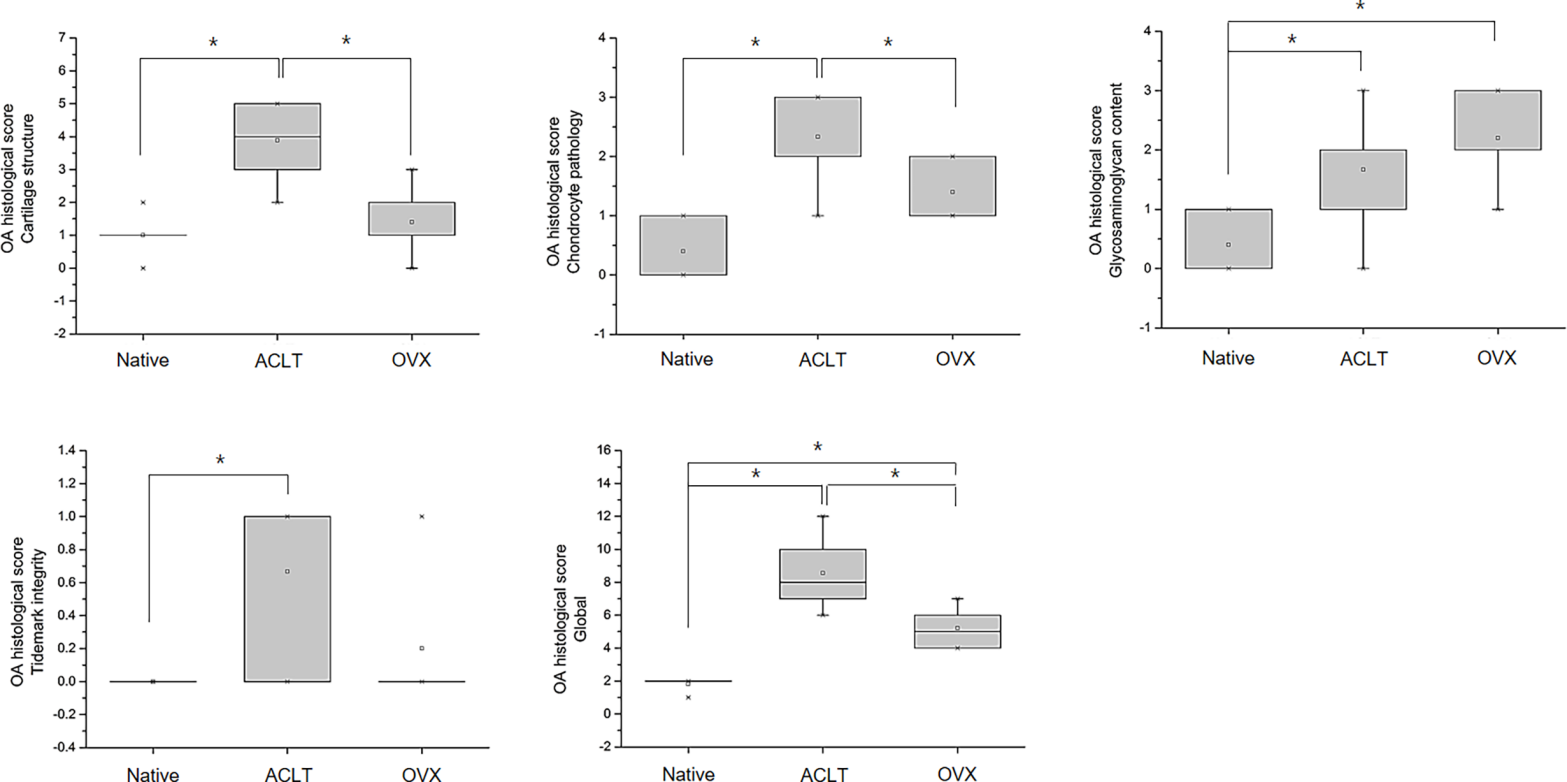
Evaluation of the modified Mankin score according to cartilage structure, chondrocyte pathology, glycosaminoglycan contents and tidemark integrity in the OA rat model. The total global score was the sum of these four scores (*P < 0.05).

**Fig 7:**
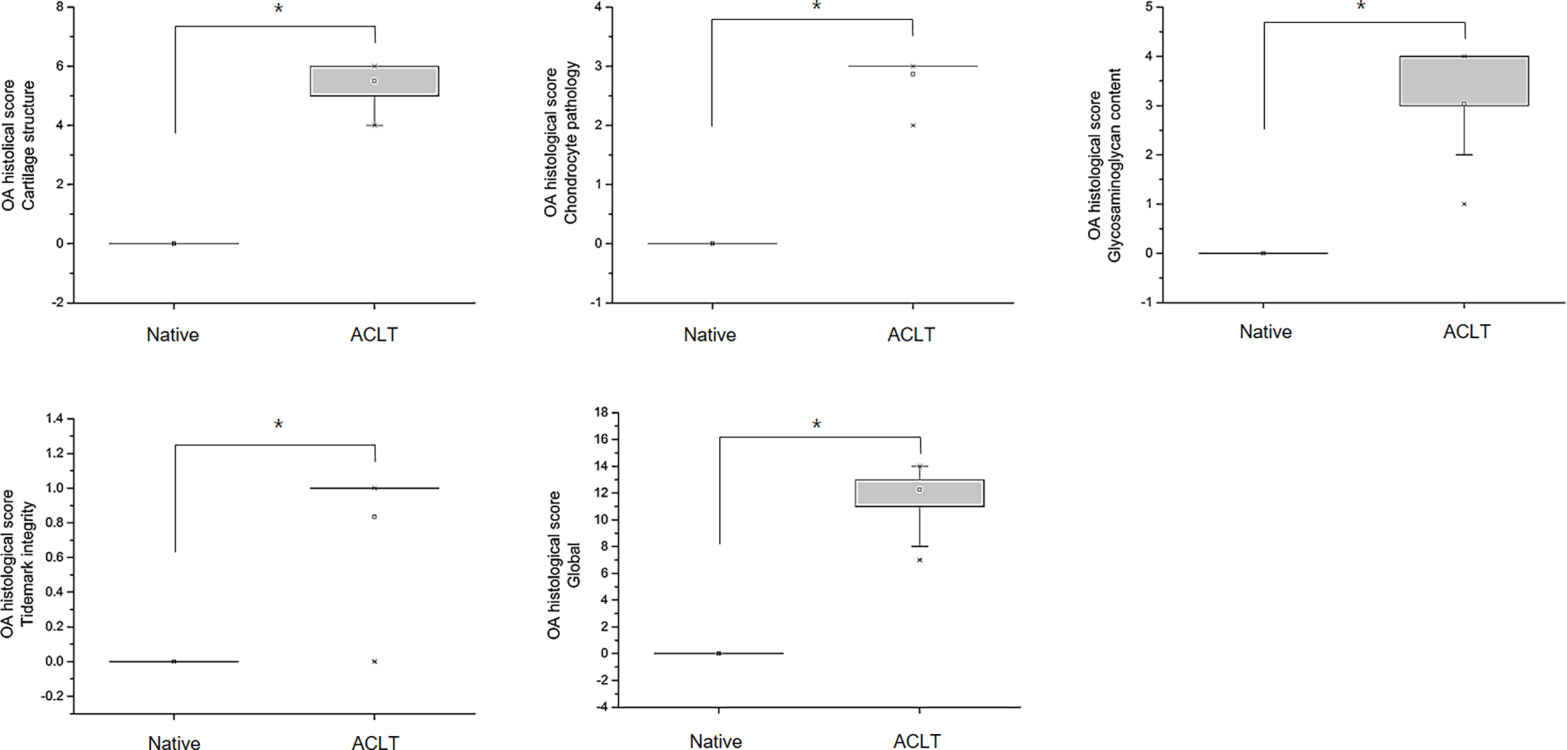
Evaluation of the modified Mankin score according to the cartilage structure, chondrocyte pathology, glycosaminoglycan contents and tidemark integrity in the OA rabbit model. The total global score was the sum of these four scores (*P < 0.05).

**Fig 8:**
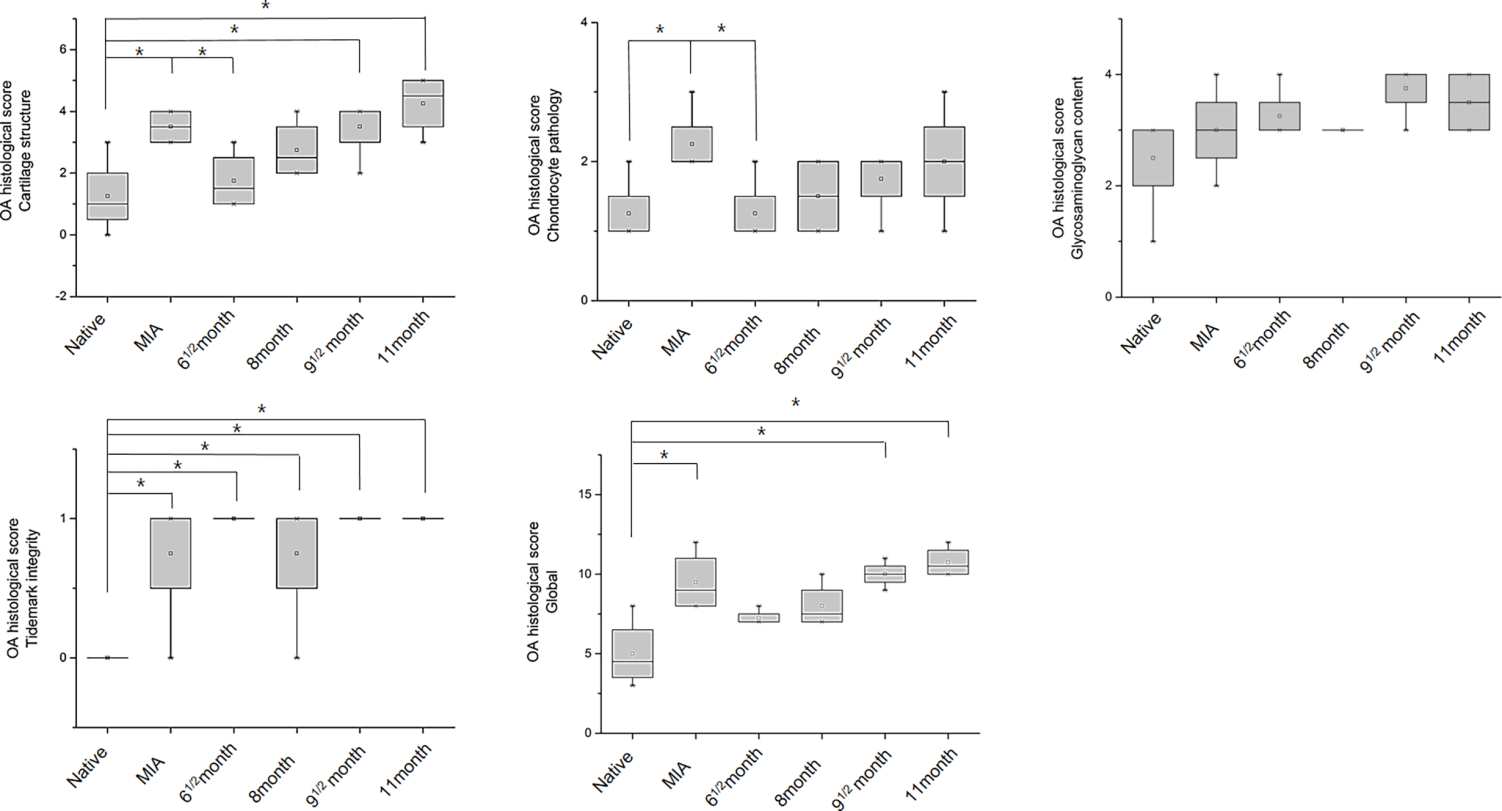
Evaluation of the modified Mankin score according to the cartilage structure, chondrocyte pathology, glycosaminoglycan contents and tidemark integrity in the OA Dunkin-Hartley guinea pig model. The total global score was the sum of these four scores (*P < 0.05).

To objectively analyze the structural changes in the histological images in a semi-quantitative manner, the modified Mankin scoring system was used in this study (Figs 6–8). The global score of the rat OA model was 1.8 ± 0.4 in the native group, 5.2 ± 1.3 in the OVX group, and 7.2 ± 0.8 in the ACLT group, which were statistically significantly different between the groups (*p* < 0.005). Likewise, the global score of the rabbit OA model was 11.8 ± 2.8 in the ACLT group, however, the score of the native group was 0 points. This result showed a significant difference between the ACLT and native group ((*p* < 0.005). In the guinea pig OA model, the global score was 5.0 ± 2.2 in the non-treated group, 9.5 ± 1.9 in the MIA group, 7.3 ± 0.5 in the 6^1/2^ month group, 8.0 ± 1.4 in the 8 month group, 10.0 ± 0.8 in the 9^1/2^ month group and 10.8 ± 1.0 in the 11 month group. The score was higher in the 11 month group than in the other groups. In these results, we identified that OA histological pathogenic changes occur in a time-dependent manner in the naturally occurring OA groups. Compared to the non-treated native cartilaginous tissues, a significant difference appeared starting at 9^1/2^ months (*p* < 0.05).

## Discussion

The various animal models including naturally occurring and surgical induced OA models examined in this study resulted in morphological changes (Table 1); however, there were differences in the progression time. In contrast, hormonally and chemically induced OA did not show any morphological changes but did show compositional changes. The results of this study showed lesion assessment and characterization using various OA animal models which can be used for tissue engineering. Although various OA disease models have been widely studied, we particularly focused on OA animal models based on a naturally occurring process. We wanted to elucidate the induction of OA as well as naturally occurring OA in rats, rabbits and Dunkin-Hartley guinea pigs without a mouse. Small animal models are widely used to research OA due to its ease of use, low cost and less time. However, mouse has very thin cartilage layer which lacks discernible radial, transitional, and superficial layers [40]. For that reasons, mouse OA model is known to difficult to induce small defects that progress slowly [41]. It commonly used for transgenic experimental models because they have ability to genetically modify or breed specific strains susceptible to OA [30]. Although small animal model warrants further testing in larger animals before clinical tests for human degenerative OA, we used rat instead of mouse as small OA model in this study.

**Table 1:** List of experimental osteoarthritis animal models used in this study.

Species	Induction method	Induction type	Characteristics
1. Rat	ACLT	Surgically induced	• Most common induced model • Rapid and reproducible progress • Permanent instability
	OVX	Surgically induced	• Related to hormone metabolism in the body • Induction osteoporotic changes by decreasing the estrogen level • Mimic the development of the OA changes in postmenopausal women
	MIA^1)^	Chemically induced	• Easily local injection method • Inhibition of glycolysis and disruption of chondrocyte metabolism • Similar characteristics with human OA in rodent cartilage
2. Rabbit	ACLT	Surgically induced	• Larger joint size than mice and rats • Rapid induce and cartilage change • Similar appearance with gross of human knee
3. Dunkin hartley Guinea pig	MIA	Chemically induced	• Easily induction method • Inhibition of glyceraldehyde-3-phosphate dehydrogenase activity
	Spontaneous	Aging, Naturally occurring	• Slowly simulating OA • Best representation of primary osteoarthritis in humans

^1)^ This model was not used in this study.

In our study, OA induced models were accomplished by surgical and chemical treatments which have advantages and are effective in terms of animal care cost, ease of handling due to rapid rate of induction. However, because primary OA leads to a slow progressive change to cartilage disorder, it was necessary to monitor how the constituents of the knee joint in the guinea pig OA model change starting from the early phase of the disease. An additional aspect of the guinea pig OA model is that the OA in that model resembles human OA [42]. Thus, the use of Dunkin-Hartley guinea pigs as an OA animal model is important for carrying out critical research. This model can be helpful in investigating human OA by examining its pathogenesis and evaluating research results in pre-clinical and clinical tests. However, the spontaneous OA model has one major disadvantage: the researcher has to wait for an injury to occur because progressive OA develops very slowly.

Micro-CT enables 3D construction imaging of cartilage tissues and provides integrated morphologic information and precise volumetric assessments. In the case of the surgically and chemically induced OA models, the morphological changes were remarkable compared to the native group (no-induction group). However, the spontaneous OA guinea pig model did not have remarkable changes as the age of the animals increased. The reason for these results could be the difference between the OA induction methods with the surgical and chemical methods resulting in rapid progression and the spontaneous method resulting in the slow progression of OA. Tokuda et al. investigated the changes in the knee joints of guinea pigs by monitoring the changes in the articular cartilage and subchondral bone from the early stage to the end stage. They investigated the incidence of spontaneous OA in the guinea pig OA model with radiological analysis. They showed that OA including osteophyte formation was indicated in nearly 30% of the total animals. Therefore, it is inferred that naturally occurring OA animal models are necessary to observe the slight minute differences using elaborate histological analysis and microscopy [38].

Although there is no current specific indicator evaluating the histologic findings of osteoarthritic specimens, histopathology has been widely used as a gold standard for assessing OA in animal models [43]. Many studies have already used macroscopic and histological evaluation of OA disease progression. Above all, the point-based grading system by Mankin is well known and used in animal models to study OA [44,45]. In this study, the histological assessment was evaluated by the general architecture and cell features with H&E staining of sections from knee joints and by Mankin’s histological grading system. We also assessed the distribution of glycosaminoglycan with alcian blue and safranin-O staining. Both alcian blue and safranin O are histochemical staining for identifying the cartilaginous structure. Alcian Blue is a dye used to detect cell chondrogenesis, and it stains the sulfated proteoglycan in cartilage tissue. Similarly, safranin-O is a basic stain which binds with proteoglycans in cartilage with a strong affinity forming an orange color. For these reasons, we observed the sulfated proteoglycan in the sectioned joint specimens using safranin O staining method instead of alcian blue staining in rat OA model. Using a modified Mankin scoring system for the guinea pig OA model, knee joint specimens were analyzed, and a significant difference was observed between the MIA model and the early spontaneous model (6^1/2^ month group and 8 month groups). However, this tendency was different for the 9^1/2^month group after naturally caring of guinea pig. The Mankin score extraordinarily increased at 9^1/2^ months for the spontaneous model which was similar to the score for the MIA model. MIA induced OA by chemically treatment results in the rapid progression of the disease to form terminal OA which could be occurring because of the strong chemical response of MIA in the animal model, while in the spontaneous model, osteoarthritic changes proceed based on natural aging without any treatment. The reason for the difference in the histological grades between these two models could be a result of the difference in the induction time of OA for the two different induction methods in the animal models.

In this study, the results were different based on the induction techniques using ACLT and OVX in the rat OA model. The ACLT group had remarkable structural destruction and compositional changes compared to the OVX group. The ACLT OA model was induced by cutting the ligaments such as the ACL and MCL; thus, these processes directly affected the knee joint by physically increasing the friction surface in the OA rat model. Conversely, the OVX OA model was induced by eliminating the ovaries and subsequently decreasing the secretion of estrogen. For these reasons, the OVX OA model is related to hormone metabolism in the body; thus, its effects are less direct than that of the ACLT OA model. Finkelstein et al. reported that the bone mineral density (BMD) is decreased in postmenopausal women which results in osteoporosis [46]. Thus, we investigated the ACLT, OVX and the combined ACLT and OVX OA models and identified whether the bilateral combined model had any significant osteoarthritic changes according to the different induction techniques described in a previous study [47]. When we induced OA by OVX surgery in the rat model, the BMD values were slightly decreased compared to the ACLT group. However, the OVX group had less cartilage damage and destruction than that of the ACLT group shown in the micro-CT images and histological analysis results. Ovariectomy induces osteoporotic changes by decreasing the estrogen level which might be associated with a low bone mineral density rather than with a qualitative visible criteria such as cartilage destruction, abnormal cartilage cell shape and distribution of cartilage extracellular matrix. Thus, a low bone mineral density would facilitate delayed bone remodeling rates and consequently osteoporosis.

Besides rats and guinea pigs, this study also investigated the OA model in rabbits surgically induced by the ACLT technique. The rabbit ACLT model is used in OA studies because rabbit knees are similar in gross appearance to human knees; however, their biomechanical function is very different. Nevertheless, the rabbit ACLT model is commonly used in early OA studies and induces rapid and severe cartilage changes as well as the destruction of the subchondral bone. Furthermore, the size of the rabbit joint is larger than that of small-sized animals such as mice and rats which has garnered interest in this species as a source of experimental models for osteoarthritic studies. In our histologic data which included H&E and Alcian blue staining, the ACLT group had incomplete cartilage shapes containing a thin cartilage layer and less chondrocytes compared to the native cartilage specimens from the rabbits. Moreover, the results from our scoring system showed a remarkably change pre- and post-surgery. Yoshioka et al. investigated an OA rabbit model induced by ACLT examining the different stages of OA progression. They found full-thickness ulcerations in articular cartilage at 8 and 12 weeks after the ACLT surgery [11]. In this study, we examined our rabbit OA model at 24 weeks post-surgery, and consequently, structural damage and cartilage destruction were definitely observed in the knee joint specimens, which also revealed significant differences between the native and OA group shown by the quantitative scoring system.

In summary, we induced OA with surgical and chemical techniques in rat, rabbit and guinea pig models. In addition, we observed the degenerative change of the knee joint based on the naturally occurring progression of OA in a guinea pig model. Although small-sized animals such as mice and rats provide advantages including low cost and easy handling, they have disadvantages such as not providing enough cartilage tissue for osteoarthritic studies. Furthermore, large sized-animals share more similarities with humans in terms of macroscopic and microscopic anatomy unlike small sized animals. To overcome this problem, more OA studies are required using large-sized animal such as canines, goats and horses for pre-clinical and clinical studies.

## Conclusion

OA is commonly accompanied by considerable morbidity involving pain and disability as chronic health problems; however, there are few treatments available. Animal models of OA are important to study the pathogenesis of cartilage degeneration and the efficacy of potential therapeutics. Therefore, we induced OA animal models using various induction methods in rats, rabbits and guinea pigs in this study. We found that these OA models had osteoarthritic changes compared to non-OA induced animals. Additionally, time dependent changes were observed in the spontaneous guinea pig animal model of OA shown by macroscopic and histologic analyses. These OA models may contribute to not only future studies on OA pathologic change but also to future studies on the clinical aspects of potential therapeutic agents for human degenerative OA.

## Supporting information

S1 Table Standards of osteoarthritis modified Mankin’s Score. (DOCX)

S2 Table Evaluation of the modified Mankin score according to the cartilage structure, chondrocyte pathology, glycosaminoglycan contents and tidemark integrity in the OA animal model. (DOCX)

## Abbreviations

OA: Osteoarthritis
ACLT: Anterior cruciate ligament transection
OVX: Bilateral ovariectomy
MIA: Sodium monoiodoacetate
ECMs: Extracellular matrices
H&E: Hematoxylin and eosin
BMD: Bone mineral density
