# Supplementary material for: Development and characterization of various osteoarthritis models for tissue engineering
Source: PLoS One. 2018 Mar 13;13(3):e0194288. doi: 10.1371/journal.pone.0194288 (PMC5849317; doi:10.1371/journal.pone.0194288)
Supplement: S1 Table — (DOCX) [file pone.0194288.s001.docx]

**S1 Table. Standards of Osteoarthritis Modified Mankin’s Score**

| **I. Cartilage structure** | |
| --- | --- |
| Normal | 0 |
| Surface irregularities | 1 |
| Pannus and surface irregularities | 2 |
| Clefts to transitional zone | 3 |
| Clefts to radial zone | 4 |
| Clefts to calcified zone | 5 |
| Complete disorganization | 6 |
| **II. Chondrocyte Pathology** | |
| Normal | 0 |
| Diffuse hypercellularity | 1 |
| Cloning | 2 |
| Hypocellularity | 3 |
| **III. Glycosaminoglycan Content** | |
| Normal | 0 |
| Slight reduction | 1 |
| Moderate reduction | 2 |
| Severe reduction | 3 |
| No dye noted | 4 |
| **IV. Tidemark integrity** | |
| Intact | 0 |
| Crossed by blood vessels | 1 |
