# Supplementary material for: Development and characterization of various osteoarthritis models for tissue engineering
Source: PLoS One. 2018 Mar 13;13(3):e0194288. doi: 10.1371/journal.pone.0194288 (PMC5849317; doi:10.1371/journal.pone.0194288)
Supplement: S2 Table — (DOCX) [file pone.0194288.s002.docx]

**S2 Table. Evaluation of the Modified Mankin score according to the cartilage structure, chondrocyte pathology, glycosaminoglycan contents and tidemark integrity in the OA animal model.**

|  | | Score Index | | | | |
| --- | --- | --- | --- | --- | --- | --- |
| Species | **Induction type** | I.  Cartilage Structure | II. Chondrocyte  Pathology | III. Glycosaminoglycan Content | IV. Tidemark  Integrity | V.  Global  Score |
| Rat | Native | 1.0 ± 0.7 | 0.4 ± 0.5 | 0.4 ± 0.5 | 0.0 ± 0.0 | 1.8 ± 0.4 |
|  | ACLT | 3.2 ± 0.8** | 2.2 ± 0.4** | 1.2 ± 1.0* | 0.6 ± 0.5* | 7.2 ± 0.8** |
|  | OVX | 1.4 ± 1.1 | 1.4 ± 0.5* | 2.2 ± 0.8** | 0.2 ± 0.4 | 5.2 ± 1.3** |
| Rabbit | Native | 0.0 ± 0.0 | 0.0 ± 0.0 | 0.0 ± 0.0 | 0.0 ± 0.0 | 0.0 ± 0.0 |
|  | ACLT | 5.5 ± 0.7** | 2.9 ± 0.3** | 3.0 ± 0.9** | 0.8 ± 0.4** | 12.2 ± 1.8** |
| Dunkin hartley guineapig | Native | 1.3 ± 1.3 | 1.3 ± 0.5 | 2.5 ± 1.0 | 0.0 ± 0.0 | 5.0 ± 2.2 |
|  | MIA | 3.5 ± 0.6* | 2.3 ± 0.5* | 3.0 ± 0.8 | 0.8 ± 0.5* | 9.5 ± 1.9* |
|  | 6^1/2^ month | 1.8 ± 1.0 | 1.3 ± 0.5 | 3.3 ± 0.5 | 1.0 ± 0.0* | 7.3 ± 0.5 |
|  | 8 month | 2.8 ± 1.0 | 1.5 ± 0.5 | 3.0 ± 0.0 | 0.8 ± 0.5* | 8.0 ± 1.4 |
|  | 9^1/2^ month | 3.5 ± 1.0* | 1.8 ± 0.5 | 3.8 ± 0.5 | 1.0 ± 0.0* | 10.0 ± 0.8** |
|  | 11 month | 4.3 ± 1.0** | 2.0 ± 0.8 | 3.5 ± 0.6 | 1.0 ± 0.0* | 10.8 ± 1.0** |

Values are expressed as means ± SD.

**P* < 0.05 versus native group, ***P* < 0.01 versus native group in each animal.
